# The Point Count Transect Method for Estimates of Biodiversity on Coral Reefs: Improving the Sampling of Rare Species

**DOI:** 10.1371/journal.pone.0152335

**Published:** 2016-03-24

**Authors:** T. Edward Roberts, Thomas C. Bridge, M. Julian Caley, Andrew H. Baird

**Affiliations:** 1 Australian Research Council Centre of Excellence for Coral Reef Studies, James Cook University, Townsville, QLD, Australia; 2 Australian Institute of Marine Science, Townsville, QLD, Australia; Instituto Español de Oceanografía, SPAIN

## Abstract

Understanding patterns in species richness and diversity over environmental gradients (such as altitude and depth) is an enduring component of ecology. As most biological communities feature few common and many rare species, quantifying the presence and abundance of rare species is a crucial requirement for analysis of these patterns. Coral reefs present specific challenges for data collection, with limitations on time and site accessibility making efficiency crucial. Many commonly used methods, such as line intercept transects (LIT), are poorly suited to questions requiring the detection of rare events or species. Here, an alternative method for surveying reef-building corals is presented; the point count transect (PCT). The PCT consists of a count of coral colonies at a series of sample stations, located at regular intervals along a transect. In contrast the LIT records the proportion of each species occurring under a transect tape of a given length. The same site was surveyed using PCT and LIT to compare species richness estimates between the methods. The total number of species increased faster per individual sampled and unit of time invested using PCT. Furthermore, 41 of the 44 additional species recorded by the PCT occurred ≤ 3 times, demonstrating the increased capacity of PCT to detect rare species. PCT provides a more accurate estimate of local-scale species richness than the LIT, and is an efficient alternative method for surveying reef corals to address questions associated with alpha-diversity, and rare or incidental events.

## Introduction

Coral reefs are one of the most diverse ecosystems on Earth [1] containing both high species richness and heterogeneity of habitats at all spatial scales [2]. For several decades, coral reefs have provided ecologists with important insights into processes that generate and maintain biodiversity, such as species richness gradients and species coexistence mechanisms (e.g. [3, 4]). A common feature of ecological assemblages is a species abundance distribution featuring a small number of common species, and many rare taxa [5–7]. These rare taxa often form the bulk of biodiversity in an assemblage, but are the most time consuming to adequately record. A high number of rare species therefore requires a large sampling effort to effectively characterize a site. This presents a significant logistical issue in high-diversity ecosystems such as coral reefs and tropical rainforests, where the number of rare and incidental taxa is very high [8]. Coral reefs in particular present additional challenges for data collection, as many reefs are remote and some habitats, such as at depth, are difficult to access.

Ecological studies of coral reefs were greatly enhanced by the advent of SCUBA diving in the 1950s, but the capacity to study reefs at depths >30 m is still limited [9]. Consequently, important questions surrounding the spatial extent, biodiversity and ecological significance of deeper reef habitats remain unresolved [10]. Overcoming this knowledge gap requires the development of new methods that enable more rapid collection of ecological data from deeper habitats. Ideally, such methods would also be broadly applicable across a range of depths and sampling regions.

Standardized methods in empirical data collection for benthic communities in marine ecosystems were developed in the 1970s primarily in conjunction with the increased use of SCUBA (e.g. [11]). The line intercept transect (LIT), adapted from terrestrial vegetation studies, has been widely used for coral reef studies (e.g. [12]). In this method, a transect line of a set length is placed along a reef, and the identification of each species under the line is recorded along with the distance it occupies. The LIT provides a precise estimate of abundance (i.e. coral cover and density), making it well suited to examination of temporal or spatial trends in the abundances of species. LITs, however, are not appropriate for all ecological questions or locations. For example, the length of time taken to complete a suitable number of replicate 10 m transects (typically ≥5) makes LITs impractical in depths >15 m, below which safe bottom times for divers become severely limiting factors for SCUBA based surveys. Furthermore, because of the time required to conduct 10 m LITs, the amount of replication achieved may result in under-sampling of rare and incidental species or events. Consequently, LITs are limited in their application according to habitat and ill equipped to address questions that require the detection of rare events or species.

A fundamental tenet of ecology is that the distribution of species is not random in time or space [13], and understanding how these non-random patterns are created and maintained is a major ecological goal [14]. The mechanisms generating patterns, such as species richness gradients, are now investigated using increasingly complex statistical analyses [15, 16], which require extensive and precise data [17]. Computationally demanding analyses, such as sample-based rarefaction, enable estimates of species richness at standardized levels of sampling effort; however, data for such analysis requires large sample sizes, consistent sampling methodology and data independence [17–19]. The logistical restrictions imposed by LITs make them ineffective for addressing these questions in most situations. Consequently, little suitable data exists, or is being collected, to investigate fundamental ecological phenomena on coral reefs using these statistical techniques.

Here, we present a novel sampling technique more suitable than LITs for estimating species richness (Alpha diversity) and abundance on coral reefs: the point count transect (PCT). The method is derived from a well-established technique in avian ecology, the point count distance transect [20, 21]. Point sampling techniques are popular for monitoring songbirds, primarily for examining species richness and diversity [22]. The detectability and mobility of different bird species is highly variable, resulting in continued refinement and calibration of this method (e.g. [20]). We adapted the point transect framework to the marine environment by conducting point counts of a constrained number of individuals at stations located along a transect. Rather than timed counts (as per the point count distance transect), we utilized point counts of a pre-determined number of colonies at each station. Although taxonomically complex, surveying corals presents fewer detectability problems (i.e. audible detection, mobility, cryptic behavior) than surveying birds, substantially reducing the main source of methodological error [23]. Moreover, standardizing the number of colonies sampled in each count controls for effort, ensuring a repeatable and efficient sampling unit. We compared the effectiveness and time efficiency of the PCT method to traditional LIT surveys for estimating species richness at the same reef site at Lizard Island, Australia. We compared 1) total species richness estimated from a standardized sample size, 2) species accumulation rate per unit effort (per additional individual, and per minute), and 3) species abundance distributions, to reveal detectability bias towards rare and incidental species.

## Methods

### Point Count Transect Survey Method

**Fig 1:**
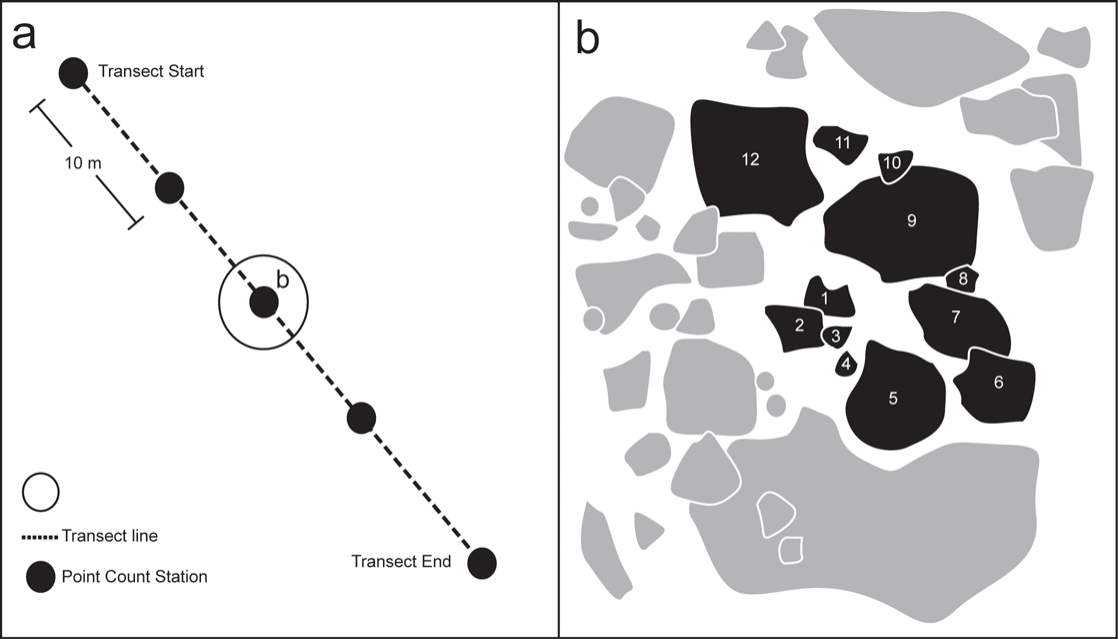
PCT Sampling Scheme. a) overview of transect with count stations, b) one count sample (12 colonies). Shaded shapes represent recorded colonies, with numbers representing the progressive sampling order. Directionality of the count progression (in this case counterclockwise) is flexible, but should be decided prior to the study.

A linear transect of a specified length (in this case 50 m) is randomly deployed within the study site, with count stations located at regular intervals (in this case every 10 m) along the transect line (Fig 1A). The transect length, and the spacing of count stations is highly flexible, depending on the research objective. For example, a study of species richness over depth could use a vertical transect up a reef slope, with count stations at bathymetric, rather than distance intervals. In that case the transect length would be variable depending on the reef profile, as would the linear distance between count stations, but the survey principle remains the same. An initial coral colony situated on a consolidated section of reef substrate suitable for coral habitation is chosen and identified at each sampling station. The nearest neighboring colony to the initial colony is then chosen as the next in the survey (Fig 1B). Successive colonies are identified such that the sampling area expands outwards in an approximately counterclockwise spiral shape from the initial colony (Fig 1B). The directionality of the expanding spiral should remain consistent, but either counterclockwise or clockwise can be chosen. As this method details reef-building coral occurrence patterns, areas known to be unsuitable for habitation, or which exclude the vast majority of species (eg. sand dunes, unconsolidated rubble banks) are not targeted. This is in contrast to existing area-based methods (eg. LIT) which often invest significant resources sampling areas of unsuitable habitat, which yields little relevant data. Additionally, the stipulation to survey suitable habitat, even when colonies are rare or absent, is an important measure of sampling effort, and represents a record of range limits, environmental filters, or other environmental factors influencing species range distributions. The requirement for types of habitat suitable for surveys can be expanded or restricted based on the research question. For a study focusing on species richness of *Acropora* spp. for example, areas of sand can be avoided, while a study focusing on *Fungia* spp. may only target sand areas. Colonies < 5 cm diameter were not recorded in this study due to difficulties consistently identifying juvenile corals to species level [24].However, the minimum size of recorded colonies will be dictated by the taxonomic expertise of the surveyor. For instance, if fragments are collected for genetic analysis, or if the locally extant species are easily differentiated, this size limit may be significantly lower. After a pre-determined number of colonies is recorded at each station (in this case 12), the surveyor moves to the next sampling station (in this case located 10 m along the transect). Twelve colonies were selected at each sampling station for this study as experience suggested that this was the maximum number reliably recorded by the observer in ~5 minutes. This value should be determined prior to the start of the survey, and be suited to the question asked. The currency in this survey method is the individual colony, grouped into count stations, which allows for the number of individuals to be chosen to suit the research question and location of the study. For instance, the research question in this case focused on time efficiency at each site, in a species rich region, so a short test revealed the maximum number of individuals reliable recorded in the chosen time limit (12 colonies in 5 minutes). In regions where coral density and/or richness is lower (such as the Caribbean, or East Pacific) a smaller number may be more suitable. Conversely, where time restrictions are not so severe, a larger number of colonies can be recorded at each sampling station. For this study, average colony densities allowed this number to be successfully recorded at each site, but to account for regions where colony densities are low, only colonies with at least part of the colony occurring within a two metre radius of the initial start colony are recorded. Colonies are countable as long as part of the colony occurs within the two metre radius. Where individual colonies extend beyond the sampling area, the size is recorded, but this is not deducted from the sampling area. If the pre-determined number of colonies cannot be found, the sampling will stop when the area is exhausted. For each colony, the species, water depth (to the nearest 0.1 m, corrected to lowest astronomical tide), maximum diameter and its perpendicular width (to the nearest 5 cm) are recorded. Species are identified *in situ* where possible, or with reference to a high-resolution image.

### Comparing the Methods

Comparative surveys were conducted along the upper reef slope of ‘Big Vickies’ reef, Lizard Island, Australia (145.44° E, 14.683° S). No permit was required from the Great Barrier Reef Marine Park Authority (GBRMPA) due to the limited impact (non-extractive) nature of the research, conducted under the accreditation of James Cook University. Only visual surveys were conducted, and no endangered or protected species were collected or manipulated. Transects to be used for both methods were laid end to end along the reef slope where there was contiguous hard substrata between 2 and 4 m depth. Nineteen replicate 10 m LITs surveys were conducted, covering the same linear reef area as the PCT while representing a sampling intensity significantly greater than the three to five transect recorded in most studies. In addition to species identity, we recorded the time taken to complete each transect. We then conducted 4 PCTs of 50 m in length (containing 6 count stations per transect at 10 m intervals) as described above overlying the same reef area. The time taken to complete each survey was recorded.

The efficiency of the two methods was compared through the rate at which new species were observed against both time invested and the number of colonies surveyed. Species accumulation curves [15] were used to compare estimates of alpha diversity from each method. Differences in sampling effort were accounted for using species accumulation curves extrapolated to a sample size of 50 samples (~600 individuals) through rarefaction using the program EstimateS [25]. Curves were used to compare the rate of increase (indicating the rate of observing new species) and the number of species recorded at a common sample size (468 individuals). The average time taken to increase the sample size by one individual was used to compare the time efficiency of each method. Species abundance distributions (SADs) were calculated to detect and display sampling bias towards or against rare species. Results are presented as mean ± 95% CI, unless otherwise stated.

## Results and Discussion

### Species Accumulation and Abundance

**Fig 2:**
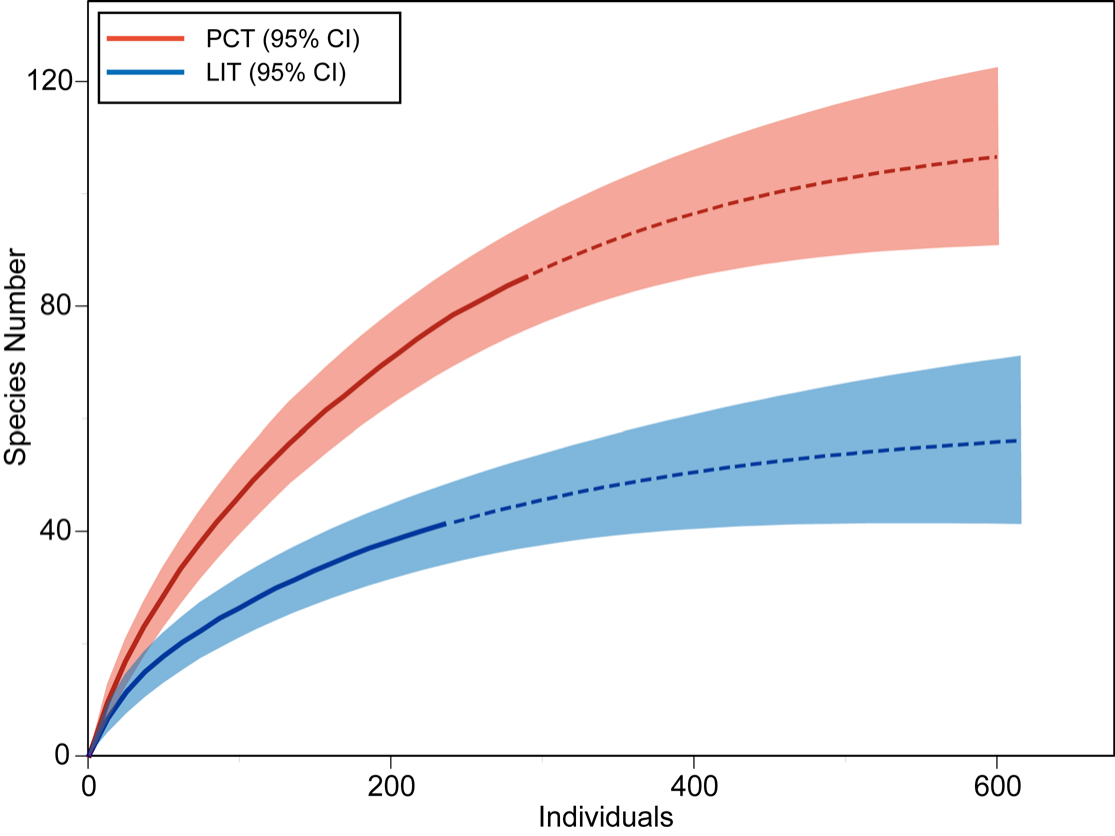
Species Accumulation Curves For PCT And LIT (by individuals added). Species richness (y axis) by number of individual colonies sampled (x axis). Solid lines represent observed species richness, dashed lines show projected species richness rarefied to ~600 individuals, with corresponding 95% CI intervals (shaded area).

**Fig 3:**
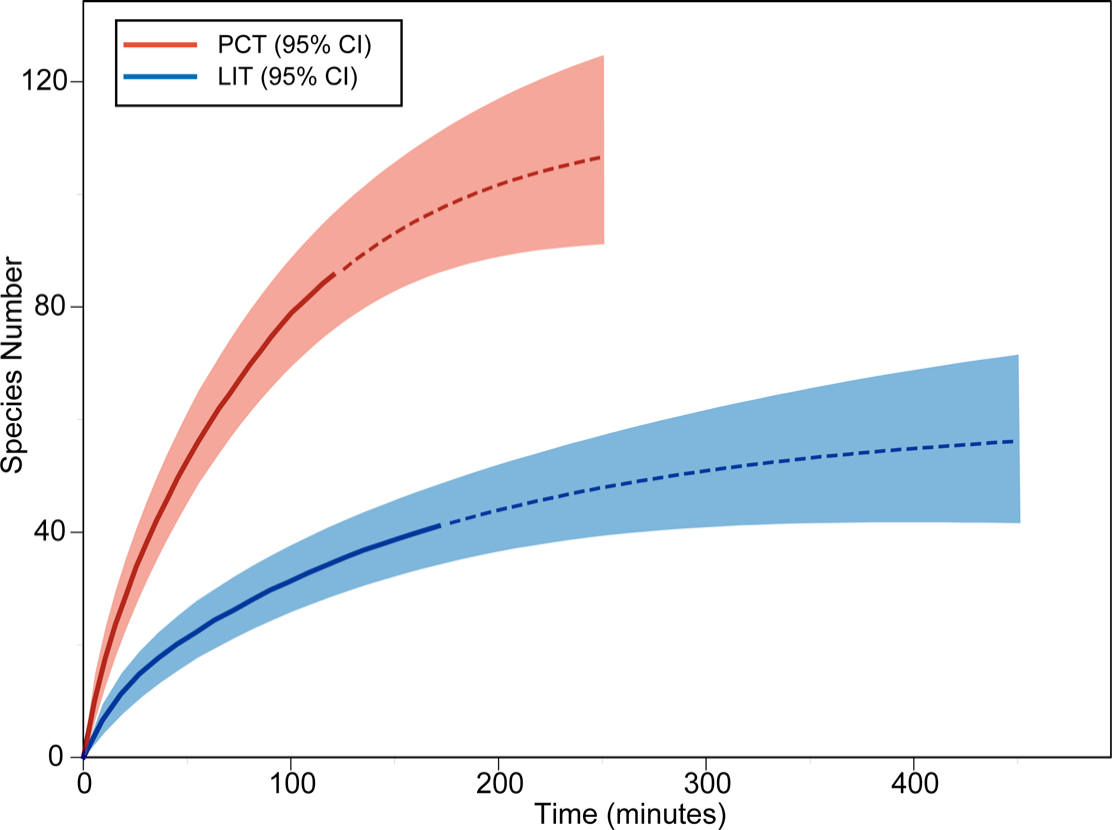
Species Accumulation Curves For PCT And LIT (by time invested). Species richness (y axis) by number of minutes invested in sampling (x axis). Solid lines represent observed species richness, dashed lines show projected species richness rarefied to ~600 individuals, with corresponding 95% CI intervals (shaded area).

A total of 234 colonies were recorded on the LITs, compared to 288 colonies during the PCTs. A mean of 12.3 colonies were recorded for each 10 m LIT, compared to the 12 colonies sampled for each station of the PCT. PCTs recorded 85 species in 120 minutes, compared to the 41 in 171 minutes for the LIT. The rate of species detection was faster for the PCT and mean estimated species richness higher for any given sample size (Fig 2). This difference was even greater when comparing species richness for any given sampling time (Fig 3). Importantly, estimates of total site species richness did not converge with the PCT species accumulation curve when extrapolated using rarefaction (Fig 2). At a comparable sample size (468 individuals), the estimated species number was substantially lower for the LIT (52.83, 95% CI: 41.13–64.53) than the PCT (100.99, 95% CI: 88.5–113.49). This disparity was even greater when time invested was accounted for (LIT: 42.85 95% CI: 35.44–50.27, PCT: 100.3 95% CI: 88.08–112.51 for 189 minutes) (Fig 3). The number of species recorded by PCT after sampling 288 colonies (83 species) was also substantially higher than the estimated total species richness after sampling 600 colonies using LIT (56 species). Although both methods showed an asymptotic accumulation curve, the projected estimates of total species richness between the methods were substantially different. Even with increased effort LITs are likely to underestimate the number of species present far more than comparable PCTs.

**Fig 4:**
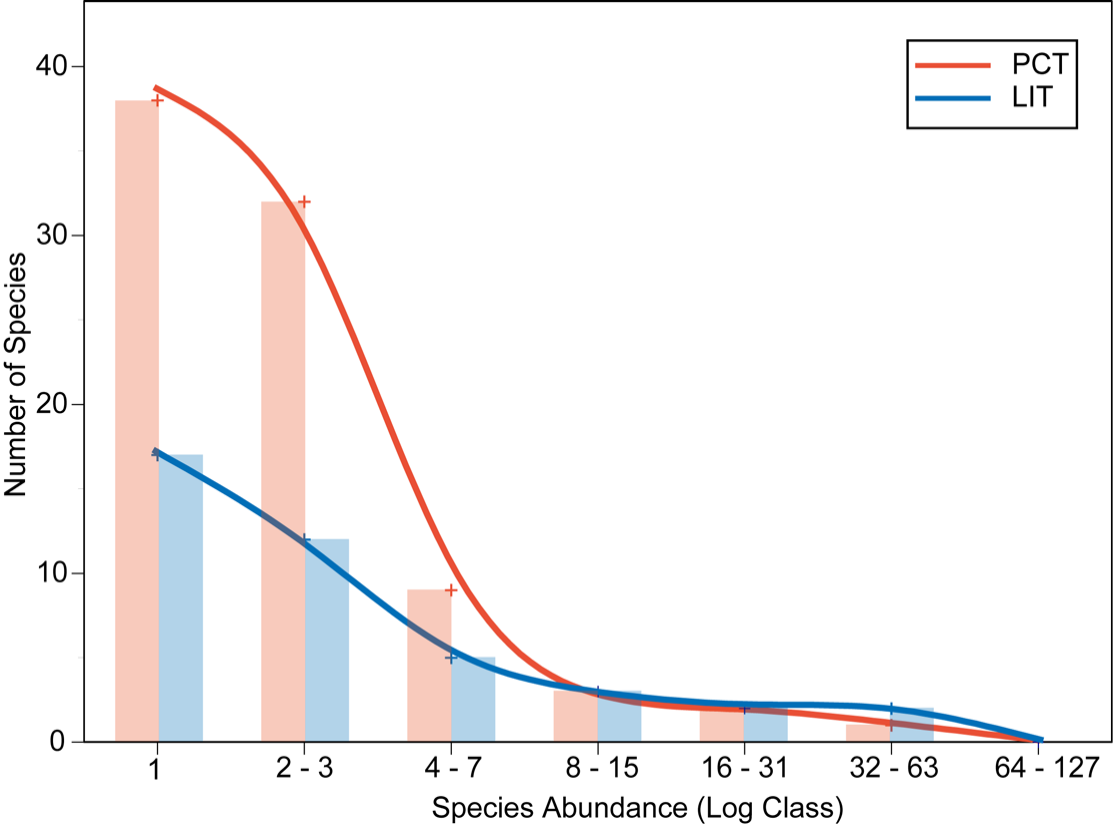
Species Abundance Distribution (SAD) Of PCT (red) and LIT (blue). Frequency bins as per Gray et al. [28] (1, 2–3, 4–7, 8–15…).

The SADs revealed that 41 of the 44 species recorded in PCTs but not in LITs were rare (observed ≤ 3 times; Fig 4). This indicates that the cause of the disparity between richness estimates was the failure of LITs to detect rare species (Fig 4). Both methods indicated similar abundances among common species, but LITs consistently failed to detect rare species even though the number of replicate transects used at Big Vickies reef (n = 19) was considerably higher than the usual number of replicates used to characterize coral assemblages at any particular site (e.g. [26, 27]). The cause of this chronic lack of detection of rare species by the LIT is likely due to the practical limitations of the method. Coral reef habitats are complex environments, with many microhabitats within a small region. The LIT method can only detect species that can be covered by a stationary line from above, and the application of the transect line is almost always unable to follow the reef contours precisely, missing most of the complex habitat. In theory, the LIT should not under-represent rare species, but the practical limitations of deploying the method in coral reefs causes errors. The real-world limitations of sampling methodologies are an important consideration, but are often overlooked in favour of theoretical justifications. Given the importance of detecting rare species for many ecological studies, we suggest that PCTs can be a more effective method of surveying coral assemblages than LITs.

The PCT was developed to assess patterns of species richness and meta-community structure along steep environmental gradients (e.g. depth) on coral reefs. These types of research questions do not require metrics of absolute abundance such as coral cover, which can be effectively obtained using LITs. As a result, the PCT represents a complementary data collection technique, rather than a replacement. The sensitivity of the PCT to rare and incidental species allows insight into the poor detection by the LIT, but emphasizes rapid capture of richness at the expense of absolute abundance measures. Using the PCT without considering its own strengths and weaknesses to a specific research question will likely result in an equally erroneous result as misuse of the LIT. Where detection of rare species is important, we propose the PCT as a robust and time-efficient method of collecting ecological data on coral reefs. This method will be particularly effective for examining questions such depth-diversity gradients, where the amount of survey time is greatly restricted. While this protocol was tested in a highly species rich habitat, with high coral abundance, it is applicable to any environment. The flexibility of the methodological framework allows for adjustment to specific systems, and questions.

Our results also highlight the importance of collecting field data using methods appropriate for the question being asked to avoid error in interpreting findings. For example, estimating species richness of a particular site using species accumulation curves requires samples to have no detectability bias towards or against any given species [17]. Bias against rare species may confound results, and can be difficult to quantify unless the extent of the bias is known. The sensitivity of such analysis to sampling error and bias is well established (e.g. [8]), yet basic errors continue to occur [6, 17].

Coral reef ecologists should continue to develop new and improved methodologies to overcome logistical constraints, and improve the precision and scope of available data. Establishing the real-world strengths and weaknesses of various methodologies enables more researchers to make a more informed decision when collecting data. Methods such as the PCT can complement existing techniques, enabling researchers to better match data collection to suit the desired analysis.

## Supporting Information

S1 File Sampling data for LIT and PCT, with EstimateS analysis outputs. (XLSX)
